# Healthy Kidney Segmentation in the Dce-Mr Images Using a Convolutional Neural Network and Temporal Signal Characteristics

**DOI:** 10.3390/s21206714

**Published:** 2021-10-09

**Authors:** Artur Klepaczko, Eli Eikefjord, Arvid Lundervold

**Affiliations:** 1 Institute of Electronics, Lodz University of Technology, 90-924 Łódź, Poland; 2 Department of Health and Functioning, Western Norway University of Applied Sciences, 5063 Bergen, Norway; eli.bjovad.eikefjord@hvl.no (E.E.); arvid.lundervold@uib.no (A.L.); 3 Department of Biomedicine, University of Bergen, 5009 Bergen, Norway; 4 Mohn Medical Imaging and Visualization Centre, Department of Radiology, Haukeland University Hospital, 5021 Bergen, Norway

**Keywords:** dynamic contrast-enhanced MRI, convolutional neural networks, kidney segmentation, pharmocokinetic modeling, perfusion quantification, glomerular filtration rate

## Abstract

Quantification of renal perfusion based on dynamic contrast-enhanced magnetic resonance imaging (DCE-MRI) requires determination of signal intensity time courses in the region of renal parenchyma. Thus, selection of voxels representing the kidney must be accomplished with special care and constitutes one of the major technical limitations which hampers wider usage of this technique as a standard clinical routine. Manual segmentation of renal compartments—even if performed by experts—is a common source of decreased repeatability and reproducibility. In this paper, we present a processing framework for the automatic kidney segmentation in DCE-MR images. The framework consists of two stages. Firstly, kidney masks are generated using a convolutional neural network. Then, mask voxels are classified to one of three regions—cortex, medulla, and pelvis–based on DCE-MRI signal intensity time courses. The proposed approach was evaluated on a cohort of 10 healthy volunteers who underwent the DCE-MRI examination. MRI scanning was repeated on two time events within a 10-day interval. For semantic segmentation task we employed a classic U-Net architecture, whereas experiments on voxel classification were performed using three alternative algorithms—support vector machines, logistic regression and extreme gradient boosting trees, among which SVM produced the most accurate results. Both segmentation and classification steps were accomplished by a series of models, each trained separately for a given subject using the data from other participants only. The mean achieved accuracy of the whole kidney segmentation was 94% in terms of IoU coefficient. Cortex, medulla and pelvis were segmented with IoU ranging from 90 to 93% depending on the tissue and body side. The results were also validated by comparing image-derived perfusion parameters with ground truth measurements of glomerular filtration rate (GFR). The repeatability of GFR calculation, as assessed by the coefficient of variation was determined at the level of 14.5 and 17.5% for the left and right kidney, respectively and it improved relative to manual segmentation. Reproduciblity, in turn, was evaluated by measuring agreement between image-derived and iohexol-based GFR values. The estimated absolute mean differences were equal to 9.4 and 12.9 mL/min/1.73 m^2^ for scanning sessions 1 and 2 and the proposed automated segmentation method. The result for session 2 was comparable with manual segmentation, whereas for session 1 reproducibility in the automatic pipeline was weaker.

## 1. Introduction

Renal function is routinely assessed by measuring serum creatinine level. Based on its value, the glomerular filtration rate (GFR) can be estimated using e.g., the Modification of Diet in Renal Disease equation [1]. Another method to determine GFR, which has recently gained popularity in the clinical setting, is the iohexol plasma clearance test. Some authors postulate that this method replaces the gold standard technique based on urinary clearance of inulin [2]. All these procedures, however, allow for quantification of GFR simultaneously for both kidneys. Hence, the dynamic contrast-enhanced magnetic resonance imaging (DCE-MRI) appears as an attractive alternative—it enables noninvasive monitoring of the filtration performance in a single kidney, while providing spatially-resolved information on tissue lesions such as focal segmental glomerulosclerosis.

In principle, the DCE-MRI examination produces a series of T1-weighted volumes acquired at multiple discrete time steps of the scanning procedure. The method consists in intravenous administration of a bolus of a gadolinium-based contrast agent (CA). While the CA bolus passes through the abdominal arterial tree, the capillary bed and tubular systems of the kidneys it effectively increases the T1 relaxation time of the penetrated tissues, thus modifying the contrast in the image. The temporal dynamics of this image signal intensity change reflects physiological conditions of kidney function and constitutes the basis for pharmacokinetic (PK) modeling of renal perfusion.

A number of the proposed PK models attempt to mathematically describe the process of blood perfusion in various organs. In regard to the kidney, most of the models assume that signal measured in a given tissue voxel is a sum of contributions originating from at least two compartments—intravascular (IV) and extracellular extravascular (EEV) spaces [3,4,5,6]. Furthermore, as in each PK model, the delivery of the gadolinium tracer through a feeding artery to the organ of interest is encapsulated by the so-called arterial input function (AIF). Practically, AIF in case of the kidney studies, is the time-course of the contrast agent concentration in the abdominal aorta [7]. By convolving the AIF with a shifting and dispersion kernel one obtains tracer concentration in the IV compartment. Eventually, the time curve of the concentration in the EEV space is proportional to the integral of the concentration in the IV compartment, optionally modulated by an exponential factor representing the outflow from the renal tubules. The proportionality coefficient, frequently denoted as $K^{trans}$, controls the rate of CA transfer from IV to EEV compartment. $K^{trans}$ multiplied by volume of the organ directly leads to calculation of GFR.

More complicated, multi-compartmental approaches exist (e.g., [8]), although their potential clinical application is questionable. The difficulty lies in the need to ensure stability of optimization of such a model parameters while fitting model curves to the observed data. Moreover, these models require segmentation of the kidney parenchyma into cortex and medulla. This requirement applies, in fact, also to two-compartment models. The classic model proposed in [3], which is based on the general Rutland–Patlak model [9,10], is applicable only to the cortex region. On the other hand, application of the two-compartment filtration model [5] to the whole kidney is acceptable for the uptake phase. If one wants to measure perfusion, the analysis must be again limited to the cortex only. In any case, one has to exclude the pelvis region which collects the contrast at the excretory phase of the examination. Therefore, automating the procedure of DCE-MRI data analysis requires not only delineation of the kidney but also labeling each renal voxel as belonging either to the cortical, medullar or pelvic class.

The problem of kidney segmentation has been tackled by many authors. Frequently, voxels are classified based on their intensity time courses. For example in [11], k-means algorithm is used to group voxels into clusters based on their signal intensity time courses. This approach was further developed in [12], where signal intensity time courses were preprocessed by the discrete wavelet transform. In the end some sort of heuristic based on both inherent clusters characteristics and relationship thereof must be employed to determine which cluster represents cortex, medulla, pelvis and background. The entirely unsupervised inference suffers from noisy input data which cannot be unambiguously classified to anatomically meaningful categories. It is especially apparent with respect to the voxels lying on borders between cortex and medulla. They are assigned to the “other parts” class which contains voxels also from outside of the kidney.

Therefore, a frequently followed strategy consists in firstly separating a whole kidney from other parts of an image. The delineated regions of interest should precisely fit kidney borders in order to get rid of all neighboring voxels. An example of such a solution are the area-under-the-curve maps (AUC), used e.g., in [13]. Those voxels in the DCE-MRI sequence which are penetrated by the tracer agent appear bright on AUC maps due to the largest area under their signal intensity time courses. It is instructive to note that this approach requires either manual or automatic post-processing in order to remove extra-renal structures, large blood vessels and urinary collecting ducts.

The coarse-to-fine segmentation strategy was also applied in [14], where the concept of Maximally Stable Temporal Volumes (MSTV) was introduced. The MSTV features allow one to recognize kidneys by detecting spatially homogeneous and temporally stable structures. Fine-grained segmentation is obtained by reducing voxels time courses to vectors of principal components, which are next partitioned by k-means to multiple clusters. Eventually, however, the obtained segmentations need to be iteratively refined in order to remove any remaining noises. Similarly, in the study described in [15], the first stage of the segmentation procedure consists in characterization of signal enhancement dynamics of the abdominal tissues. It is observed that medulla, unlike cortex, liver or spleen, exhibits steady increase in signal intensity occurring later in the acquisition sequence. After medulla voxels are identified, dilatation is performed followed by the GrubCut algorithm to create renal masks. Fine-tuning is achieved by classifying voxels with a pre-trained random forest classifier. Voxels are characterized by their respective image intensities in selected time frames of the dynamic sequence as well as their location within the ROIs constructed in the first stage.

Although both MSTV- and GrubCut-based contributions seem to produce satisfactory results for multiple data sets, they are rather conceptually complex algorithms, unavailable in either commercial or open-source software. As such, they cannot be easily adopted by the clinical community.

On the other hand, recent advances in convolutional neural networks architectures and wide availability of their software implementations make deep learning methods particularly attractive in application to segmentation of biological tissues in medical images. Effectiveness of CNNs in versatile scenarios of MRI data analysis is thoroughly discussed in the comprehensive review of [16]. More specifically, in [17] various network architectures, i.e., fully convolutional network [18], SegNet [19], U-Net [20], and DeepLabV3+ [21], have been tested for semantic segmentation of prostate cancer in T2-weighted MRI. Anatomical MR images were also analyzed in [22]. The authors developed a custom CNN architecture to automatically outline polycystic kidneys. Another interesting approach has been presented in [23], where deep learning was employed for direct inference of brain perfusion maps from a DCE-MRI sequence without explicitly fitting a PK model to measured signals.

There have been several published studies on application of CNNs to segmentation of kidneys in computed tomography images [24,25]. However, DL-driven segmentation of the kidney in contrast-enhanced MRI datasets still remains an unexhausted topic. As an exceptional example of the published works specifically dedicated to renal DCE-MRI examinations one can mention the paper of Haghighi et al. [26]. They constructed and trained a cascade of two CNN networks. The input to this cascaded structure is a 4D DCE-MR image. The first network roughly localizes the left and right kidney in the image, whereas the second one is responsible for precise delineation of renal borders. However, no further segmentation into cortex or medulla was performed since only the whole renal parenchyma was used to calculate GFR.

In light of the above considerations, the approach presented in this paper is a response to the need for an automatic algorithm that on one side would accurately recognize the kidneys compartments within the DCE-MR image, while on the other side being relatively easy to reproduce by any research team. Therefore, we propose to:

1. use a CNN architecture for semantic segmentation of the kidney parenchyma,
2. describe parenchymal voxels with feature aggregates derived from the transformation of the signal intensity waveforms by the principal components analysis (PCA),
3. discriminate cortex, medulla and pelvis regions through classification of the extracted PCA feature vectors.

Details of the employed algorithms are presented in Section 2. In Section 3, we evaluate the segmentation results and how the designed solution affects stability of estimated PK parameters. While performing this assessment, we also hypothesize that it is possible to construct a universal computing scheme that is capable of producing correct segmentations for new subjects based solely on historical data. We believe that such a scheme could be distributed widely and implemented in clinics with no or little effort to adapt to custom image acquisition protocols. In order to achieve this goal, we have validated the proposed computational process in a leave-one-subject-out manner. We have created a series of segmentation and classification engines, each trained on a different cohort of patients. Then, a given engine has been tested on a subject unseen during the training phase. Discussion of the obtained results is given in Section 4.

## 2. Materials and Methods

### 2.1. Mri Data

The experiments and algorithms presented in this paper were designed using a set of 20 measurement sequences obtained from 10 healthy volunteers. MRI examinations were performed on a 1.5 T unit (Siemens Magnetom Avanto, Erlangen, Germany). The data acquisition protocol embraced imaging each subject twice, 10 days apart, using the contrast-enhanced 3D spoiled gradient echo method (TE/TR/FA = 0.8/2.36/20 ${}^{\circ }$ ms/ms/-, in-plane resolution = 2.2×2.2 mm${}^{2}$, slice thickness = 3 mm, acquisition matrix = 192×192, number of slices = 30). Contrast agent (0.025 mmol/kg of GdDOTA) was administered intravenously at 3 mL/s flow rate. Each measurement sequence consisted in 74 frames acquired at 2.3 s time intervals.

To reduce motion artifacts, the imaging protocol was designed to acquire images on breath holds. Firstly, eight baseline pre-contrast volumes were acquired during 18-s breath-hold period. At 7 s after the Gadolinium injection, the participants were instructed to hold the breath for 26 s for motion-free, first pass perfusion. Next, during the filtration phase breath-hold periods were shortened to 13 s and interleaved with 26 s of free-breathing. In order to ease breathing, subjects nasally received oxygen at a flow rate of 1 L/min.

The remaining kidney motion was corrected in post-processing by executing b-spline registration on each DCE-MRI sequence. For that purpose, we employed the B-splines algorithm implementation from the Insight Toolkit (ITK) software library [27]. In every sequence, we selected a reference frame corresponding to a midpoint of the perfusion phase and then used it as a fixed volume that every other (moving) volume was matched to. B-splines registration was performed fully automatically, i.e., no fiducial points were marked over tissues of interest. Additionally, the procedure was launched in a multi-stage configuration. In each stage, various settings of grid size and subsampling rates were used. For a detailed interpretation of these parameters the reader is referred to the ITK documentation. In short, they allowed performing registration of images at different scales—starting from coarse matching and then refining the outcome.

For the need of algorithms design and evaluation, all volumes were manually annotated by a radiologist with expertise in MR urography. In each time frame, a left and right kidneys were delineated. Then, in two frames linked to perfusion and excretion phases, parenchymal voxels were assigned to cortex, medulla or pelvis.

In between of the examinations, volunteers underwent the iohxol clearance procedure. Subjects were administered a dose of 5 mL of iohexol (300 mg I/mL; Omnipaque 300, GE Healthcare). Then, ground-truth GFR values were determined to enable validation of image-derived perfusion estimates.

Participants were instructed to refuse from alcohol and high-protein meals, avoid excessive physical exertion, be normally hydrated at least 2 days before examination, and have no caffeine on the examination day. To ensure comparable examination conditions between scanning sessions and the iohexol clearance test, it was also recommended to maintain regular eating times and diet. All volunteers gave their written informed consent for participation in the study which was approved by the Institutional Review Board at the Haukeland University Hospital Bergen, Norway.

### 2.2. Overview of the Segmentation Pipeline

**Figure 1:**
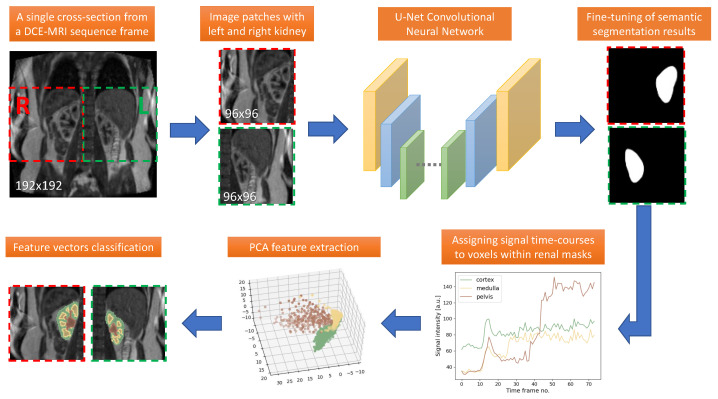
Overview of the Designed Segmentation Pipeline.

The proposed segmentation pipeline is visualized in Figure 1. The initial coarse segmentation is accomplished by a fully convolutional neural network of the U-Net structure. This step was performed on subsequent two-dimensional cross-sections of a single volumetric image from the DCE-MRI sequence. This image corresponds to the frame of the highest signal enhancement in the cortex region, when the partitioning of the renal parenchyma into cortex and medulla is clearly visible.

We assumed that a single cross-section can be divided into left and right sides, each of 96-pixel width. On a given side, it is possible to centrally locate an image patch of 96-pixels height covering entirely one kidney. In this way, we ensured that left and right kidneys are segmented and processed separately. Currently our method required this assumption to hold during the recall phase of the network operation. While training, it was sufficient if an image patch contained at least part of the renal parenchyma. Moreover, the 96×96 patch size was adjusted to the in-plane resolution of the DCE-MRI data available in this study and should be modified under different acquisition configurations.

Coarse segmentation may sometimes require additional refinement to reject small extrarenal clusters of falsely segmented pixels. In our algorithm, the connected components were identified and only the largest one was passed on to the next stage. Additionally note that although our semantic segmentation step was performed in 2D, it was applied to all cross-sections of a given volume. The analyses which came next were accomplished voxel-wise so that effectively GFR was calculated from all nephrons distributed in the 3D cortex ROI.

Thanks to image registration in the time domain, kidney masks generated for one frame could be applied to all other frames of the dynamic series. Thus, renal voxels were prescribed feature vectors composed of MRI signal intensity values measured in subsequent time points. In order to obtain more general characteristics of the signal dynamics, we extracted feature aggregates using PCA transform. The 20 most informative aggregates were selected to describe each renal voxel. Eventually, a classifier trained to discriminate between temporal characteristics of cortex, medulla and pelvis regions assigned a voxel to an appropriate category.

The rationale behind the proposed two-stage approach was to ensure that final recognition was based upon maximally confident ground-truth annotations. If it were a CNN to segment out cortex from medulla and pelvis, exact target masks would have to be created and the annotator would need to make a decision where the actual boundary between the various tissues lies. In many cases, this was not trivial due to the partial volume effect. Hence, such a decision, and consequently the trained network model, could be biased towards a unique observer experience. Alternatively, we decided to train a classifier using the signals only from unambiguous locations, as described below. Later, during the forward-pass inference, it was the algorithm’s responsibility to objectively discriminate voxels belonging to any disputable regions.

Finally, all computational units of the proposed procedure—CNN and classifier model, as well as the PCA transformation matrix—were obtained for a cohort of patients independent from the currently processed data set. In the following subsections, we provide the implementation details of the individual modules of the algorithm.

### 2.3. Cnn for Semantic Segmentation

Among the available U-Net variants, we employed the implementation published at [28]. Since it differed from the architecture described in the original paper [20], the specific characteristics of this structure, adjusted to the needs of our study, is summarized first.

#### 2.3.1. Network Architecture

**Figure 2:**
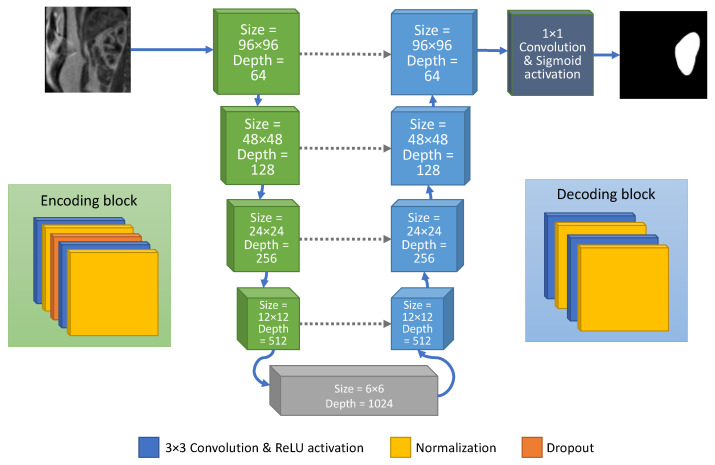
U-Net architecture of the convolutional neural network implemented for semantic segmentation of kidneys in the DCE-MR images.

The U-Net convolutional neural network was originally developed for segmentation of neuronal structures in electron microscopic stacks and proved effective in numerous other biomedical applications. As said, the input to our model was a 2D grey-level image—a cross-section patch of a single 3D DCE-MRI volume. The size of the patch was adjusted to 96×96 pixels (see Figure 2).

The characteristic feature of the U-Net is that it contains two symmetric parts—a contractive and an expansion path. The goal of the contractive path is to encode image pixels’ intensity patterns by performing convolution with a series of 3×3 filters of trainable weights. Filters outputs activate the main processing components of the network—the neurons called rectified linear units (ReLU). They allow for modeling non-linear relationships between image features and the output segmentation map. Thus, the encoding stage can be compared to a process known in digital image processing as feature extraction. It is followed by the max-pooling operation which down-samples the feature maps.

Contraction is repeated four times to extract image descriptors on various scale levels. Each level is, in fact, formed by a block composed of two convolutional layers, each followed by a batch normalization layer, which maintains constant mean and standard deviation of the output embeddings within a given batch. Thus, batch normalization ensures that features with low intensity dynamics possess equal importance as those whose range is larger. The convolution and normalization layer pairs are separated by the dropout layer, which randomly sets 20% of the input nodes to 0. This mechanism, active only during the training phase, prevents the network from overfitting [29].

The output of the last down-sampling block, referred to as bottleneck, is passed on to the expansion or decoding path. It is built up from the same number of up-sampling levels as the contractive part and its main task is to recover the original spatial resolution. In this study, up-sampling was realized by transposed convolution. Each decoding block was also composed of two pairs of convolutional and batch normalization layers. In contrast to the encoding blocks, however, no dropout mechanism was inserted in-between. Moreover, the high-resolution feature maps extracted in the down-sampling path not only fed the subsequent encoding layers but they were also concatenated to the inputs of the decoding layers at the respective levels of the up-sampling path. These additional connections helped the decoding blocks to restore kidney segments localization more precisely.

As can be seen in Figure 2, encoding blocks consisted of an increasing number of convolutional filters as going deeper down in the contractive path. Starting from 64 filters in the first two convolutional layers, the number of feature maps reached 1024 in the bottleneck, being doubled at each down-sampling level. On the contrary, the number of filters in the expansion path was divided by the factor of two on each upward step. As a result, the final feature map again had the depth of 64.

The output of the last up-sampling block was connected to a convolutional layer with a 1×1-size filters. It performed pixel-wise convolution of the filter kernel with a 64-element feature vector and then submitted the result to an output activation function. In our design, a sigmoid activation was used since the final decision was binary—a pixel belonged to renal parenchyma or background.

#### 2.3.2. Training

Network weights were initiated to a random state by the method of He et al. [30]. The training process was conducted on image patches cropped from the DCE-MRI volumes, each containing a single, left or right kidney cross-section. As described above, 96×96-pixel image patches were extracted from volumes of the DCE sequence corresponding to the perfusion phase, i.e., time frames of the maximum signal contrast between cortex and medulla. In order to increase the number of training images, for each study we actually selected three such time frames—the one with maximum signal enhancement in the cortex region, one preceding and one succeeding time frame. In each image volume, a single kidney was visible on 12 slices on average. It gave approximately 1440 training patches.

**Figure 3:**
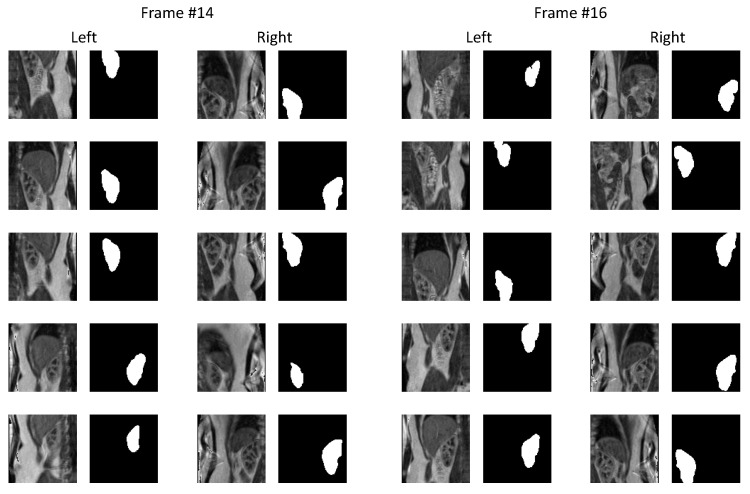
Examples of training image patches extracted from left and right kidneys from two time frames of Subject 1. Data augmentation was realized by image flipping in horizontal direction and vertical shifting of patch location relative to image center.

Although U-Net networks can usually cope with small training samples, we decided to further enlarge the data set through data augmentation. This was accomplished by picking 10 different vertical positions of the image patch and by randomly mirroring it in the horizontal direction. While selecting patch positions, we made sure that it embraced a sufficiently large portion of the image center containing significant fragment of the renal parenchyma (see Figure 3). Overall, the number of images available for training reached the value of 13,964. One third of the training images were separated for the validation purposes.

We trained 10 different CNN models, one for every patient. While building a model dedicated to a given subject, its corresponding image patches (irrespectively of the examination session) were removed from the training and validation sets and used only for testing. Weights of the network were updated using the stochastic gradient descent algorithm with the constant learning rate = 0.01 and momentum = 0.99. The loss function chosen to optimize was the binary cross-entropy criterion, defined as

(1) $$H=-\frac{1}{N}\sum_{i=1}^{N}y_{i}\log\left(p(y_{i})\right)+\left(1-y_{i}\right)\log\left(1-p(y_{i})\right)$$

where *N* is the number of voxels, $y_{i}$ is the true voxel label, and $p(y_{i})$ is the network prediction that *i*-th vector indeed belongs to class $y_{i}$, with $0\leq p(y_{i})\leq 1$. Additionally, in order to monitor the quality of kidney segmentation over training epochs, we calculated the Jaccard coefficient, hereafter designated as IoU (intersection-over-union)

(2) $$IoU=\frac{\sum_{i=1}^{K}y_{i}\wedge y_{i}^{pred}}{\sum_{i=1}^{N}y_{i}\vee y_{i}^{pred}},$$

where *K* designates the number of pixels in a processed slice and $y_{i}^{pred}$ is the predicted pixel category. Here, categories were Boolean-valued and a pixel was labeled *True* if it belonged to the kidney, *False* otherwise. In the case of each subject, the optimization algorithm was run for 50 epochs. The stored model corresponded to the epoch with the minimum score on the loss function obtained for the validation data set.

### 2.4. Classification of Kidney Voxels

#### 2.4.1. Feature Extraction

Differentiation of voxels representing particular renal compartments could be based on raw signal intensity time courses. We propose, however, to transform signal waveforms, i.e., vectors of 74 temporal features, into the space of reduced dimensionality using principal component analysis (PCA). The purpose of this transform is not only to decrease the complexity of the resulting classification model but also to extract a more general characteristics of the kidney tissue, representative for various subjects. Moreover, even in the same clinical unit, DCE imaging can be performed in a sequence that, although covering a similar time range, has a different temporal resolution. Hence, a decision-making system that accepts a uniform feature pattern, by using PCA transformation object as an adapter, can be applied to variable-length input data vectors.

We presumed that the extracted PCA components should explain at least 90% of the original data set variance. In order to fulfill this requirement for every subject, at least 20 feature aggregates had to be constructed. As we observed, larger number of components did not lead to higher classification accuracy.

#### 2.4.2. Feature Vectors Classification

Assignment of renal voxels to cortex, medulla or pelvis is performed by a classifier trained in the supervised manner. In our approach, historical data serve as patterns for building appropriate decision rules, later applied to new studies. We have tested three classification algorithms to find the best scheme across all the subjects. The methods examined included logistic regression, support vector machines and XGBoost decision trees. In the following, we recall the characteristics of the employed algorithms and describe how the training data were prepared.

**Figure 4:**
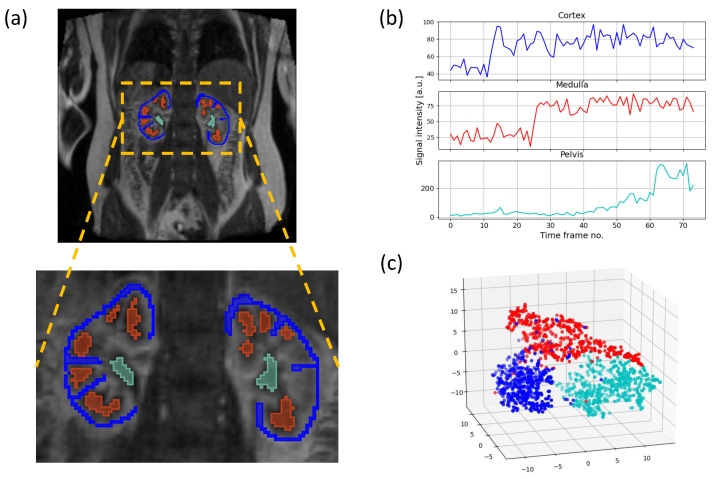
Preparation of training data for supervised learning of classifiers: (**a**) ROI placement in a DCE-MRI frame; (**b**) signal time courses assigned to corresponding ROI voxels; (**c**) three-dimensional visualization of PCA feature vectors representing cortex (blue), medulla (red) and pelvis (magenta) ROIs. The visualization was obtained by transforming 20 PCA features using t-SNE method.

**Train and test data sets.** The training vectors were acquired from regions of interest manually annotated in the respective parenchymal locations. The annotations were made only in voxels whose membership was unambiguous (see Figure 4a,b), thus letting a trained classifier to decide about the dominating tissue category in case of voxels partially filled with various compartments. The number of training vectors collected from the 20 available examinations exceeded the value of 60,000. This data set was partitioned into 10 folds, each containing data vectors from all but one subject, left apart for testing purposes. In a given fold, the class distribution was approximately as follows: cortex—58%, medulla—31%, pelvis—11%. In order to give classifiers a chance to learn to discriminate categories with equal accuracy, in each training fold the subsets representing cortex and medulla were resampled to match the size of the pelvis category. On average, the training set after resampling embraced over 16,000 vectors per fold. In a given training fold, data from both examination sessions were included. On the other hand, the testing folds contained 600 to 4800 vectors depending on the patient and examination session. Classifiers were evaluated using the balanced accuracy score calculated on the test sets.

**Logistic regression.** The logistic regression classifier models the probability that a feature vector belongs to one of two categories. The algorithm fits a linear function to the training data and the result of the regression equation for a given data point is submitted to the logistic transform

(3) $$p\left(y_{i}|x_{i}\right)=\frac{1}{1+\exp(-w_{0}-w^{T}x_{i})}$$

where $p(y_{i}|x_{i})$ denotes the probability of a class $y_{i}$ given a data point $x_{i}$ with $y_{i}\in \left\{0,1\right\}$, whereas the weight vector w together with the intercept $w_{0}$ determine the fitted regression line.

As such, Equation (3) applies to binary classification problems. Therefore, in case of three renal regions, either three one-versus-rest classifiers must be built, or a multinomial regression model is fit [31]. In our experiments we use the latter variant. Parameters w of the linear model are found by minimizing the log-loss cost function with L2 regularization term:

(4) $$L=-\frac{1}{N}\frac{1}{k}\sum_{i=1}^{N}\sum_{c=1}^{K}y_{i}\log\left(p_{i,c}\right)+\frac{1}{2}ww^{T}$$

where in our study K=3 and $p_{i,c}$ is the predicted probability that *i*-th vector belongs to class *c*. The above optimization problem was solved with the Stochastic Average Gradient (SAG) descent algorithm [32] implemented in the Scikit-Learn package [33]. We chose SAG due to its recommendation for large data sets and support for L2 regularization.

**Support Vector Machines**. Support vector machines (SVM) constitute a class of algorithms which construct a maximum margin hyperplane discriminating different categories [34]. The decision about category membership of a vector x is determined by the sign of the hyperplane equation

(5) $$y\left(x\right)=b+\sum_{\alpha_{i}\neq 0}\alpha_{i}y_{i}x_{i}\cdot x,$$

where *i* denotes the index of a training example, $x_{i}$ is a corresponding feature vector and $y_{i}$ is its true class label. While fitting this model to training data, a constrained quadratic optimization problem is solved. As a result, a set of non-zero Langrage multipliers $\alpha_{i}$ is found, which along with their respective support vectors $x_{i}$ and the shift parameter *b* determine location and orientation of the searched boundary.

A dot product in (5) can be replaced by a kernel function to enable application of SVM to non-linear problems. The kernel trick implicitly transforms the feature vectors to a space of higher dimension, in which it becomes possible to determine the separating hyperplane. Figure 4c shows the distribution of the training vectors subset for one of the participants. This visualization was obtained by transforming the data from the space of 20 PCA feature aggregates into a space of three dimensions using the t-distributed stochastic neighbor embedding (TSNE) method [35]. It can be seen, that although linear separation of cortex, medulla and pelvis classes is plausible, the separating boundary may be better modelled by some non-linear function. In our experiments, the best results were ensured by the radial basis function kernel

(6) $$k\left(x_{i},x_{j}\right)=\exp\left(-\gamma ∥x_{i}-x_{j}{∥}^{2}\right),$$

where ∥·∥ denotes the $\ell^{2}$-norm. Since in real data sets, perfect separation of the classes is rare, the optimization criterion allows—through an additional method parameter usually designated with letter *C*—allows a certain number of data points to violate the decision boundary. The γ and *C* parameters contribute conversely to the complexity of the SVM model. Therefore, we tuned their values using the exhaustive grid search algorithm in a five-fold cross-validation experiment. The best results were reported for γ=0.05 and C=1.

**XGBoost decision trees.** The concept of extreme gradient boosting (XGBoost) was introduced by Chen and Guestrin [36] to facilitate training of an ensemble of classification and regression trees (CART). In contrast to classic random forests, the learning algorithm formalizes the regularization mechanism and usage of versatile objective loss functions. Thanks to these advantages, it has recently proved effective in numerous machine learning problems involving large, high-dimensional data sets.

Construction of a XGBoost ensemble is based on the strategy of so-called additive training. At each step *t* a new tree is added to the model which minimizes the overall loss function

(7) $$L^{\left(t\right)}=\sum_{i=1}^{N}l\left(y_{i},{\hat{y}}_{i}^{\left(t-1\right)}+f_{t}\left(x_{i}\right)\right)+\Omega \left(f_{t}\right),$$

where ${\hat{y}}_{i}^{\left(t-1\right)}$ is the class label predicted by the ensemble constructed thus far, *l* measures the error between the predicted and the true label $y_{i}$, whereas $f_{t}$ corresponds to a tree structure of *T* leaves, which assigns each *i*-th data vector a score $w_{q(x_{i})}$, with *q* being a function that allocates data point $x_{i}$ at a given tree leaf. Both *T* and weights $w_{j}$ define the regularization term

(8) $$\Omega \left(f\right)=\gamma T+\frac{1}{2}\lambda \sum_{j=1}^{T}w_{j}^{2},$$

where γ and λ parameters control the impact of the tree size and leaves scores on the penalty value. In the configuration used in our study, γ and λ were set to 0.1 and 0.9, respectively. Moreover, the number of trees in the ensemble was equal to 10, and the maximum allowed depth of a tree was 4. Eventually, we used the *soft-max* function as an objective criterion *l*.

### 2.5. Pharmacokinetic Modeling

Apart from evaluating the segmentation results directly with the usage of IoU coefficient, we also compared image-derived GFR values against the ground-truth iohexol-based measurements. For the purpose of GFR determination we employed the kidney-specific two-compartment filtration model (2CFM) [5]. This PK model decomposed renal tissue into the intravascular (IV) and extracellular extravascular (EEV) spaces and assumed no tubular outflow within the modeling period. Although the model could be used to estimate renal perfusion either in cortex or whole renal parenchyma, only the prior region had to be considered while calculating the GFR.

Independently from the considered renal region, in order to fit the model to a measured signal S(t), a respective mean image intensity time-course must be converted into the concentration waveform $C_{tissue}\left(t\right)$. We accomplished it using the transformation described in [37] adjusted to the gradient echo sequence. On the other hand, $C_{tissue}\left(t\right)$ described by the 2CFM model is governed by the equation

(9) $$C_{tissue}\left(t\right)=K^{trans}\int_{0}^{t}C_{p}^{kid}\left(\tau \right)d\tau +v_{p}C_{p}^{kid}\left(t\right),$$

with

(10) $$C_{p}^{kid}=C_{p}^{art}⊗\;g\left(t\right)=\int_{0}^{t}C_{p}^{art}\left(t-\tau \right)g\left(\tau \right)d\tau ,$$

where $C_{p}^{art}$ denotes the arterial input function, $v_{p}$—plasma volume fraction, and $C_{p}^{kid}$—CA concentration in the blood plasma. The first term in (9) represents the CA concentration in the EEV space, whereas the second term covers the concentration in the IV space obtained by convolving arterial input function with the vascular impulse response function (VIRF), defined as

(11) $$g\left(t\right)=\left\{\begin{array}{cc} 0 & \;t<\Delta \\ \frac{1}{T_{g}}e^{-\frac{t-\Delta }{T_{g}}} & \;t\geq \Delta \end{array}\right..$$

As such, VIRF models the delay and dispersion of the AIF relative to the CA flow through the capillary bed. Variables $T_{g}$—the dispersion time constant, and Δ—the delay interval, together with the volume fraction $v_{p}$ and transfer constant Ktrans form the complete set of the 2CFM model parameters. Their estimation is usually performed in the non-linear least squares (NLLS) curve-fitting procedure. The Trust Region-Reflective method [38], employed in this study, is one of the possible optimizers used to numerically solve the NLLS problem. In contrast to down-simplex methods, it allows to set constraints upon parameter values and thus ensure that the final estimates fall into physiological range. The constraints presumed in our study are listed in Table 1.

We implemented the algorithm for optimization of the 2CFM model in the custom software written in Python, available for download at [39]. The algorithm was launched for each patient using the cortex regions either obtained automatically by the above-described segmentation pipeline or annotated manually. In any case, the AIF was determined automatically using our method published earlier in [40].

### 2.6. Statistical Analysis

One of the goals of automating the process of DCE-MR image segmentation is to ensure stable and repeatable perfusion estimates. Hence, we performed the Student’s *t*-test for the related (repeated) samples to verify the null hypothesis of equal GFR means estimated on two examination events. Moreover, we calculated the repeatability coefficient defined as [41]

(12) $$CoV=\frac{\sqrt{2}\sigma_{\mathrm{diff}}}{\mu_{\mathrm{pool}}}\cdot 100\%,$$

where $\sigma_{\mathrm{diff}}$ is the standard deviation of the differences between GFRs made on the same subject, and $\mu_{\mathrm{pool}}$ denotes the mean of all measurements. The quantity $\sqrt{2}\sigma_{\mathrm{diff}}$, also referred to as single measurement standard deviation ($\sigma_{\mathrm{sm}}$), is an estimate of the standard deviation of differences in pairs of potentially many consecutive measurements [42]. In addition to repeatability assessment, reproducibility of image-based GFR estimation was evaluated using Bland–Altman plots. Eventually, the results of analysis achieved for the automatically found renal segments were compared against manual annotations.

## 3. Results

**Figure 5:**
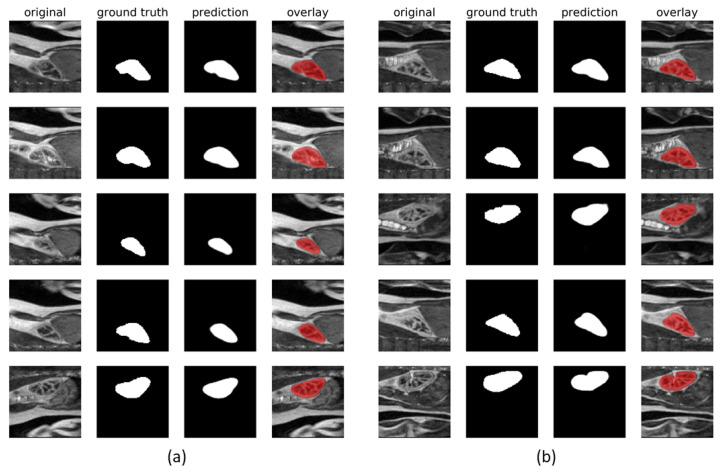
Examples of output segmentation masks compared against manual annotations for Subjects 1 (**a**) and 5 (**b**).

**Figure 6:**
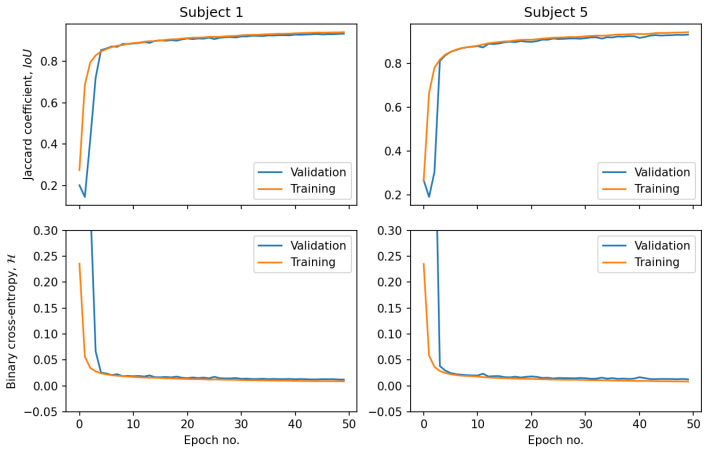
Evolution of the loss function and validation metric over the training epochs for Subjects 1 and 5.

Figure 5 shows example outputs of the semantic segmentation network for two of the participating subjects along with the ground-truth annotation masks. The corresponding training processes are visualized in Figure 6, which plots the evolution of the loss and evaluation metrics for both the training and validation sets. One may observe, that after a first few epochs, IoU curves for the training and validation sets lay close to each other and the network did not enter the overfitting state. Simultaneously, there was a monotonic decrease of the loss function, which proved that the network gained the generalization capability. The IoU coefficients of similarity between manual annotations and the automatically found kidney regions obtained after removal of the extrarenal islands disconnected with the main segments, are collected in Table 2. The mean IoU for all subjects and studies = 0.94 and it appeared laterally indifferent.

In the next stage, parenchymal voxels were classified into separate renal compartments. Comparison of the three tested classifiers is presented in Table 3. It evaluates each method with respect to three metrics—balanced accuracy, recall and precision. The latter two were determined for each renal category separately. The balanced accuracy metric was obtained as the average of true positive rates obtained for particular classes. All presented scores were mean values over 20 test subjects. The three tested classifiers achieved similar performance in terms of the true positive rates (approximately 95%). Overall, however, it was the SVM that exhibited the best balanced accuracy (96%) and also gained larger than the other methods capability to avoid false positive detections. It was particularly apparent for the pelvis region, where precision = 92% against 89% offered by logistic regression and XGBoost classifiers.

High rates of classification accuracy translated to equally good segmentation results (Table 2), which were again assessed using the Jaccard coefficient. This time, however, it was calculated as the sum of the IoUs determined for each region separately and then weighted by its support, i.e., the number of ground-truth voxels representing a given class. In order to enable comparison with the other works, we recalculated the obtained Jaccard rates to Dice coefficients ($F_{1}$-score) with the formula

(13) $$F_{1}=\frac{2IoU}{1+IoU}$$

**Figure 7:**
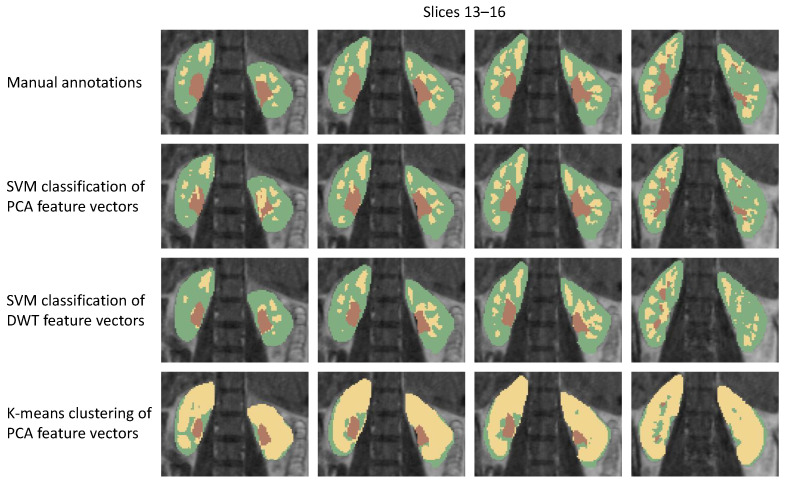
Comparison of segmentation results obtained by the proposed method with ground truth annotations and two alternative approaches postulated elsewhere (Subject 2, MR session 1).

Using our algorithm, we achieved the mean Jaccard coefficient for the cortex class in the left kidney equal to 93.2%. In case of the other regions the IoU equated to approximately 91%, except for the pelvis class in the left kidney where it dropped to 90.1%. The quality of fine segmentation can be visually confirmed by analyzing examples of the kidney decomposition into regions shown in Figure 7. In order to enable the assessment in wider context, we also performed segmentation with two alternative methods proposed in the literature. The first one consists in extracting DCE signal characteristics using the discrete wavelet transform (DWT) with the Daubechies-4 wavelet, as postulated e.g., in [12]. Then we classified DWT coefficient vectors by the help of SVM algorithm. In the second compared method, PCA feature vectors were clustered using k-means algorithm (with k=3), as described in [14]. This second approach failed to properly distinguish between cortex and medulla. Majority of voxels representing both regions were embraced in a common cluster and only pelvis was recognized as an autonomous part of the parenchyma on most of the cross-sections. In contrast to the results obtained by clustering, the regions produced in the supervised manner using DWT-based voxels description are more accurate. However, the number of false recognitions is apparently higher than in case of PCA feature vectors. The obtained balanced accuracy score for the SVM classifier was only 78% in this case. See the last two rows of Table 3 for the quantitative comparison of these alternative partitioning schemes with Xgboost, linear regression and the best in our study SVM/PCA algorithm.

**Figure 8:**
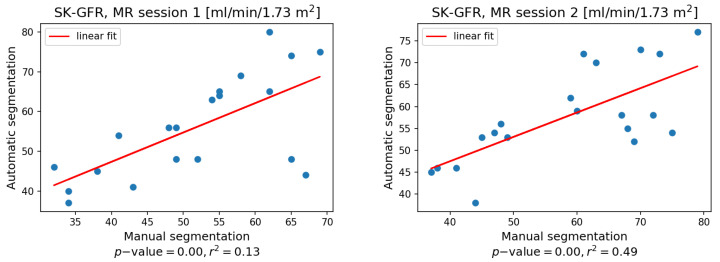
Comparison of single kidney GFR estimates obtained based on mean signals calculated in manually or automatically annotated cortex regions.

The outcomes of the segmentation stage were used to determine the mean signals in the renal cortex. This signal was then fitted to the 2CFM pharmacokinetic model. Figure 8 presents how single-kidney glomerular filtration rates (SK-GFR) obtained in this way correspond to the relevant scores derived using the manual annotations. The mean SK-GFR values across all subjects and studies as obtained after automatic and manual segmentation are similar (56 versus 55 mL/min/1.73 m^2^) and the observed difference is statistically insignificant (T-statistic = 0.75, *p*-value = 0.46). Additionally, the linear fit between the two types of measurements proves their good correspondence with $r^{2}=0.13$ and 0.49 for MR examination sessions 1 and 2, respectively.

**Figure 9:**
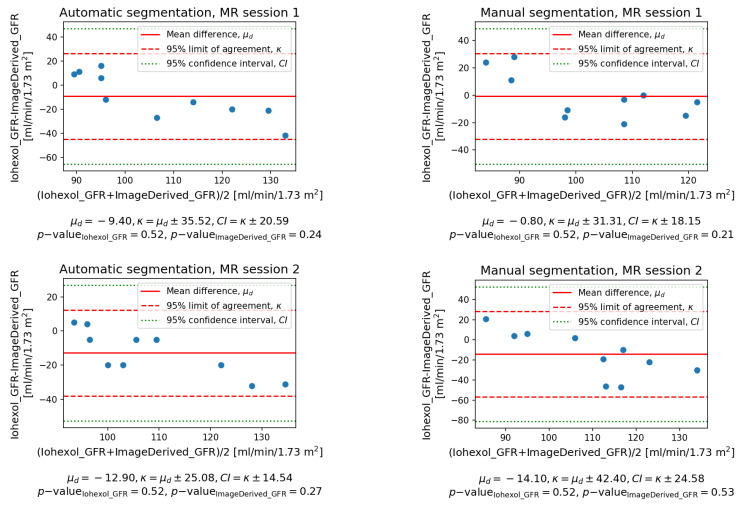
Bland–Altman plots of agreement for automatically (**left**) and manually (**right**) determined kidney segments. Measurements were evaluated against normality using Shapiro–Wilk test.

The Bland–Altman plots shown in Figure 9 allow to assess the agreement of the total GFRs with the ground truth iohexol-based rates. Prior to application of the Bland–Altman method, normal distribution of the measurements was confirmed using Shapiro–Wilk test. The obtained *p*-values are given in the relevant plot legends. The mean difference $\mu_{d}$ for the MR examination session 1 in terms of absolute values was smaller for manual segmentations (−0.8 against −7.4 mL/min/1.73 m^2^). In the case of session 2, the values of $\mu_{d}$ were consistent between segmentation methods, although the agreement with the reference method was weaker. The proposed algorithm, however, appeared to perform slightly better (−12.9 versus −14.1 mL/min/1.73 m^2^). Based on the obtained results, it could not be decided which segmentation approach ensured narrower limits of agreement. In the case of session 1, the comparison favored manual segmentation (κ = 31.3 versus 35.5 mL/min/1.73 m^2^). However, the narrowest limits of agreement and confidence intervals across all experiments were obtained for session 2 and automatic labeling (κ = 25.1 mL/min/1.73 m^2^). Both segmentation approaches led to repeatable results (see Table 4) with the coefficient of variation equal to 30.2% in the worst case (manual segmentation, left kidney). Apparently, automatic segmentation ensured better stability with coefficients of variation twice as low as in the case of manual counterparts. The *p*-values obtained in Student’s *t*-tests for the related samples were all above confidence level α=0.05 showing insufficient evidence against the null hypothesis of there being no significant difference between the observed means of SK-GFR measurements. However, the *p*-value calculated for the left kidney and manual segmentation was relatively small. Further investigation, potentially involving a larger sample, is necessary to confirm or reject repeatability of the measurements in this case.

## 4. Discussion

The primary goal of this study was to design an efficient method for kidney segmentation in DCE-MR images. We combined the concepts partially proposed in previously published works into a uniform computational framework. It embraces coarse semantic segmentation of kidney parenchyma, PCA transformation of MR signal time-courses to create voxels numerical representation and then enable their classification to produce fine-segmentation of renal tissue into cortex, medulla and pelvis. The proposed approach leads to accurate results, enabling kidney recognition at the rate of 94% in terms of Jaccard coefficient. Segmentation of particular renal compartments can be achieved with IoU between 90% and 93% (96–95% of Dice coefficient), depending on the tissue type.

When referring to other published results, the obtained ratios are either in good accordance or slightly lower. In [14], Dice score for the healthy kidney segmentation was reported at the level of 99% (cortex), 98% (medulla) and 96% (pelvis). However, it was also shown for simulated data that image noise may significantly degrade the accuracy to 82–85%. Since imaging protocols used therein and in our study differed both with respect to spatial and temporal resolution, the observed discrepancies in the range of 1–2% may be attributed to various noise levels in our studies. Comparable scanning conditions were used in one group of pediatric patients in [15]. The mean $F_{1}$-score observed therein was equal to 93% for the whole kidney, and 86% for the renal cortex. In [12] clustering-based segmentation was evaluated using accuracy scores. The average obtained results were: 88%—cortex, 91%—medulla, and 98%—pelvis. These values can be collectively referred to the balanced accuracy score which takes into account the size of a given segment. Thus, our SVM model appears to outperform the clustering-based approach offering classification accuracy, as well as recall rates at the level of 96% even for the largest cortex region. Eventually, the CNN network devoted to recognition of the whole kidney described in [26] showed lower performance than our U-Net design, achieving $F_{1}$-score = 91.4% for the normal test subjects.

The observed differences in segmentation results should also be viewed in light of the algorithm training-recall configuration. In contrast to some alternative approaches [11,14], our strategy is patient-independent. Once the semantic segmentation and classification models are built, they are applied to new studies, not encountered in the training set. Hence, patient-specific features cannot guide the recognition mechanism and may not fit the trained model, decreasing the overall accuracy rate. Moreover, as discussed above, due to the partial volume effect persisting on the borders between cortex, medulla and pelvis, there remains some dose of uncertainty regarding the reference manual segmentations which must be taken into account while analyzing the reported metrics. Although the SVM classifier was trained on carefully selected, tissue-distinctive ground-truth signals, validation data, which must embrace all renal voxels, may still suffer from subjectivism of the observer annotations.

Apart from the observed power to produce accurate segmentation results, our strategy possesses one advantageous characteristic. Since we use a supervised classifier to assign image voxels to appropriate kidney regions, there is no need for a separate labeling step. In case of clustering, it is necessary to provide some heuristic for interpreting the actual category of each cluster, if automation of the whole procedure is to be ensured.

**Figure 10:**
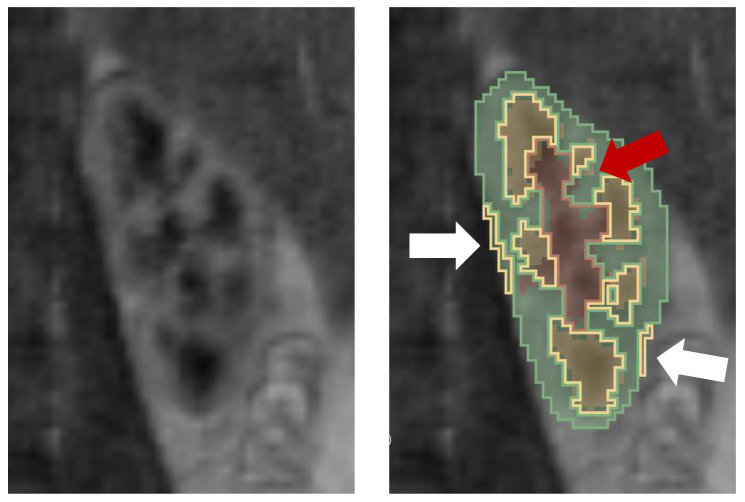
Cross section of the left kidney (Subject 1, examination session 1) and its corresponding segmentation result (solid border lines) overlaid on the manual annotation (semi-transparent fill). White arrows indicate false classifications made by the proposed method potentially due to erroneous image registration. Red arrow shows an example of a segmentation error due to partial volume effect.

As illustrated in Figure 10, there are mainly two sources of discrepancy between automatic and manual annotations. Firstly, the border between cortex and medullary pyramids is ambiguous and the corresponding voxels are partially filled with both kinds of renal tissue. While the automatic method tends to include such voxels in the cortex class, they were frequently designated as medulla by the expert. On the other hand, even the manual segmentation is not consistent in this respect which proves the difficulty in arbitrarily deciding about the class of voxels where the partial volume effect is dominant. Secondly, false detections can be observed e.g., on the external cortical edges. These effects can be attributed to the misregistration of images in the time domain. The applied b-spline registration method was not optimized for the need of this study and remains the topic of our future investigation.

The conducted experiments also showed that the proposed segmentation algorithm improves repeatability of image-based SK-GFR estimation. The calculated coefficients of variation amounted to 14.5% and 17.5% for the left and right kidney, respectively. In the case of manual annotations we obtained CoV = 30.2% and 29.4%. These results have to be assessed with respect to natural GFR variation caused by independent factors, such as diet and time of the day. It is expected that serum creatinine level, which correlates well with GFR, may achieve a variation degree of up to 10% [43]. Hence, CoV of approx. 15% indicates good repeatability of the procedure which utilizes automated kidney segmentation. Additionally, the obtained repeatability metrics fall into the values range presented elsewhere, e.g., in [44] CoV = 32% and 27%, whereas in [13], CoV = 17.5% and 15.4% for the left and right kidneys, respectively. In the latter study, however, CoV was calculated only as the ratio of $\sigma_{\mathrm{sm}}$ and the mean of all measurements, without the additional factor of $\sqrt{2}$. If this correction factor is reflected, their reported CoVs become 24.7% and 21.8%, which are fairly close to our findings.

There can be raised three limitations of our study. Firstly, the assumed partitioning into image patches may incline the semantic segmentation network to learn kidney locations only near one of the patch edges. Hence, in further development of our models it will be studied how the algorithm performs if the acquisition field of view is configured differently. Secondly, the segmentation algorithm was designed using healthy subjects only. It can have especially significant consequences in case of fine segmentation of the renal parenchyma. Adjusting the classification model to the diseased kidneys may require extending the number of classes to more than just three categories (cortex, medulla and pelvis) in order to reflect diverse temporal characteristics of renal tissue lesions. Thirdly, in order to calculate GFR, we fitted the 2CFM model to the mean DCE signal estimated in the segmented cortex. Due to the partial volume effect, both manual and automatic segmentation may classify some voxels as medullary, although they contain renal glomeruli. Ignoring such voxels may be one of the reasons for observed discrepancies between image-derived and iohexol-based GFR measurements. In order to overcome this problem, some authors [5] propose to use whole kidney ROI to make sure that all voxels contributing to renal filtration are included in the PK model fitting process. We found, however, that this approach leads to remarkable overestimation of GFR for the data sets available in our study. Hence, in the future we plan to apply deep learning-based super-resolution techniques and attempt to achieve more precise annotations of cortical voxels.

Moreover, a full 3D approach to semantic segmentation should be exploited. It can be expected that a neural network capable of processing whole MR volumes would produce more precise kidney annotations thanks to additional depth information. However, the problem in this study had to be reduced to two dimensions due to relatively small number of studies available for training. Decomposition of volumetric images into 2D cross-sections allowed to significantly increase the training data set.

Eventually, as described previously, the proposed approach was validated in the leave-one-subject-out fashion. There were effectively, 10 independent neural network and classifier models created. A legitimate question is how these models could be applied to new subjects from outside the sample available in this study. One option would be to create an ensemble and introduce a voting mechanism. Alternatively, a new segmentation scheme could be trained (embracing both coarse and fine-grained steps) based on the entire 10-subject sample. Its performance with regard to new data sets should be comparable to above-presented results.

## 5. Conclusions

To conclude, in this paper we demonstrated a computational framework for supporting quantitative assessment of kidney perfusion by providing an automated way of renal compartments segmentation. The obtained accuracy results prove reliable operation of the designed method. Moreover, in our experiments, alternative approaches to discriminate cortex, medulla and pelvis segments, based on wavelet transforms and clustering algorithms perform less effectively. Repeatability of SK-GFR measurement based on automatically found segments improves when compared with the outcomes of manual processing and also stays in good agreement with other published results. The designed segmentation method allows for increased objectivism of the image-derived perfusion parameters and also potentially faster diagnosis of renal impairments. These findings bring closer the clinical application of DCE-MR imaging as a routine method in kidney diagnostics. Finally, in order to facilitate this shift from research to application domain, we make our software framework for pharmacokinetic modeling available at [39]. The repository includes also the scripts for DCE signal-based feature extraction and classification. 

## Figures and Tables

**Table 1:** Parameter constraints presumed in 2CFM model fitting experiments.

Parameter	Min	Max
$K^{trans}$ [1/min^−1^]	0.05	0.40
$v_{p}$ [mm^3^]	0.2	0.9
Δ [s]	1.0	3.5
$T_{g}$ [s]	0.02	–

**Table 2:** Mean (and standard deviations) of IoU and $F_{1}$-scores over all subjects and MR sessions.

	IoU		$F_{1}$-Score	
Region	Left	Right	Left	Right
Kidney	0.941 (0.012)	0.940 (0.014)	0.970 (0.006)	0.970 (0.007)
Cortex	0.932 (0.042)	0.908 (0.066)	0.964 (0.023)	0.950 (0.038)
Medulla	0.912 (0.057)	0.913 (0.049)	0.953 (0.032)	0.954 (0.027)
Pelvis	0.909 (0.037)	0.901 (0.072)	0.952 (0.021)	0.946 (0.040)

**Table 3:** Classification metrics averaged over the testing sets—subjects 1–10, MR sessions 1 and 2.

Classifier	Balanced Accuracy	Cortex		Medulla		Pelvis	
		Recall	Precision	Recall	Precision	Recall	Precision
Logistic	0.943	0.939	0.963	0.947	0.931	0.942	0.889
XGBoost	0.938	0.940	0.955	0.936	0.930	0.938	0.887
SVM/PCA	**0.956**	**0.951**	**0.970**	**0.954**	**0.941**	**0.962**	**0.919**
SVM/DWT	0.784	0.926	0.692	0.703	0.811	0.722	0.801
K-means	0.527	0.846	0.584	0.118	0.067	0.617	0.863

**Table 4:** Evaluation of repeatability of SK-GFR measurements obtained after manual and automatic kidney segmentation.

Segmentation Method	Automatic		Manual	
Side	Left	Right	Left	Right
Mean difference [mL/min/1.73 m^2^]	−0.8	−2.7	−7.2	−6.1
$\sigma_{\mathrm{sm}}$ [mL/min/1.73 m^2^]	8.3	9.9	15.7	17.0
CoV [%]	14.5	17.5	30.2	29.4
*p*-value	0.69	0.28	0.08	0.16

## Data Availability

DCE-MR images used in this study cannot be made available because the written consent signed by the participants did not cover the agreement for public dissemination of the acquired data.
